# Supplementary material for: Comprehensive germline genomic profiles of children, adolescents and young adults with solid tumors
Source: Nat Commun. 2020 May 5;11:2206. doi: 10.1038/s41467-020-16067-1 (PMC7200683; doi:10.1038/s41467-020-16067-1)
Supplement: Supplementary file 1 — Supplementary Information [file 41467_2020_16067_MOESM1_ESM.pdf]

Supplementary Information

Comprehensive germline genomic profiles of children, adolescents, and young adults with solid tumors

Sara Akhavanfard, Roshan Padmanabhan, Lamis Yehia, Feixiong Cheng, Charis Eng

Supplementary Table 1 Demographic and clinical characteristics of patients

	CCF			PCGP				SJLIFE			
DNA Type	Germline			Germline				Germline			
Sequencing Assay	Whole Exome Sequencing			Whole Exome Sequencing				Whole Exome Sequencing			
#Total Patients	50			193				1269			
Age Group	Children	Adolescent	Young Adult	Children	Adolescent	Young Adult	Unknown	Children	Adolescent	Young Adult	Unknown
Female/Male	16/15	4/8	0/7	64/62	5/4	NA	14/13*	520/505	58/85	5/8	NA
Average Age of onset	7.8 ± 4.2	17.7 ± 1.2	22.3 ± 2.9	6.4 ± 4.7	16.5 ± 1.1	NA	NA	6.1 ± 4.5	16.9 ± 1.3	22.2 ± 2.5	NA
All Solid Tumors (1507)	31	12	7	126	9	0	53	1025	143	13	88
Central Nervous System (323)	1							265	23	2	32
Wilms Tumor (207)	1		1					188	2		15
Neuroblastoma (190)	2		1	21	2		25	135			4
Rhabdomyosarcoma (134)	3			26	2		1	85	12	1	4
Osteosarcoma (129)	8	4	2				2	70	38	3	2
Retinoblastoma (98)								84			14
Ewing's Sarcoma (95)	3	2	1					55	25	4	5
Soft Tissue Sarcoma (93)	6	1	1					63	16	2	4
High Grade Glioma (80)	2	3		61	5		9				
Germ Cell Tumor (74)	1							56	11	1	5
Low Grade Glioma (24)	1	1					15	7			
Adrenocortical Carcinoma (22)		1		18			1	2			
Carcinoma (14)								7	7		
Giant Cell Tumor (3)									3		
Renal Cell Carcinoma (3)			1					2			
Low Malignant Potential Renal Tumors (2)	2										
Basal Cell Carcinoma (1)								1			
Paraganglioma (1)									1		
Other Solid Tumor (10)								3	4		3
Non-Malignant Tumor (4)	1							2	1		

CCF, Cleveland Clinic Foundation; PCPG, Pediatric Cancer Genome Project; SJLIFE, St. Jude Life Cohort.

* Gender data were not available for all the patients in this group

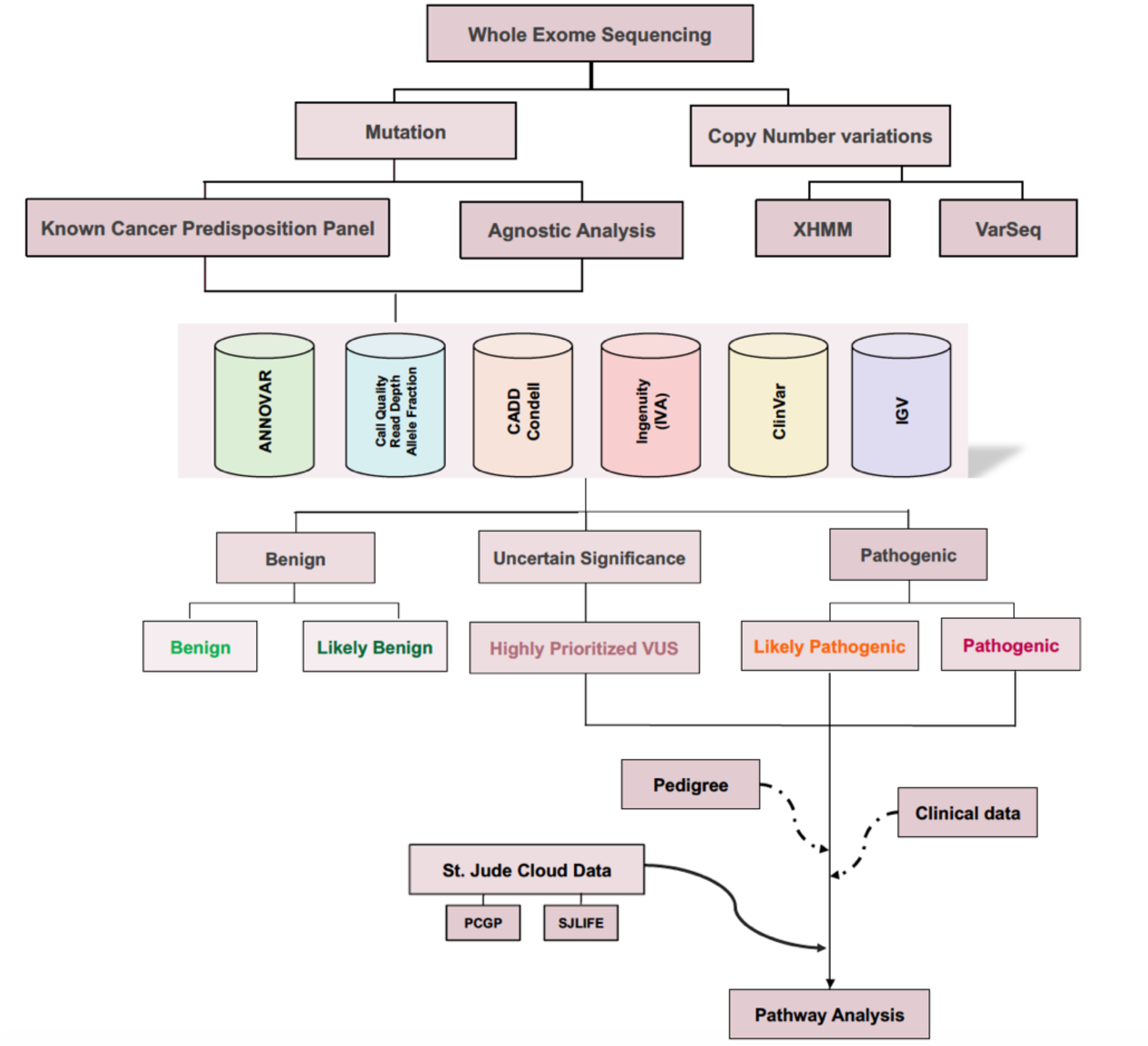

Supplementary Figure 1 Analysis Workflow. Abbreviations: XHMM, eXome-Hidden Markov Model; IGV, Integrative Genomic Viewer; PCGP, Pediatric Cancer Genome Project; SJLIFE, St. Jude Lifetime

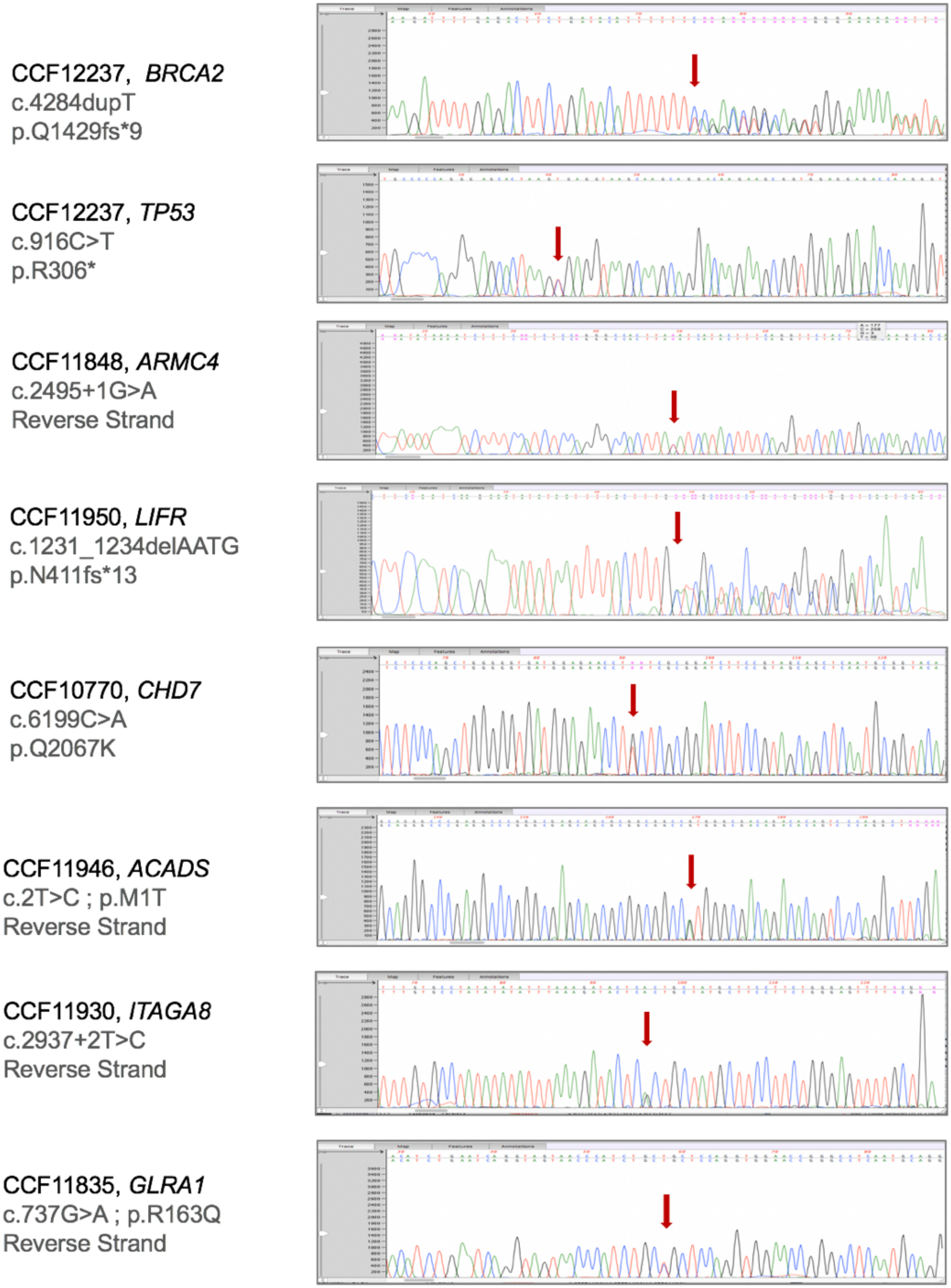

Supplementary Figure 2 Examples of Sanger sequencing confirmations of KCPG and candidate variants in CCF series

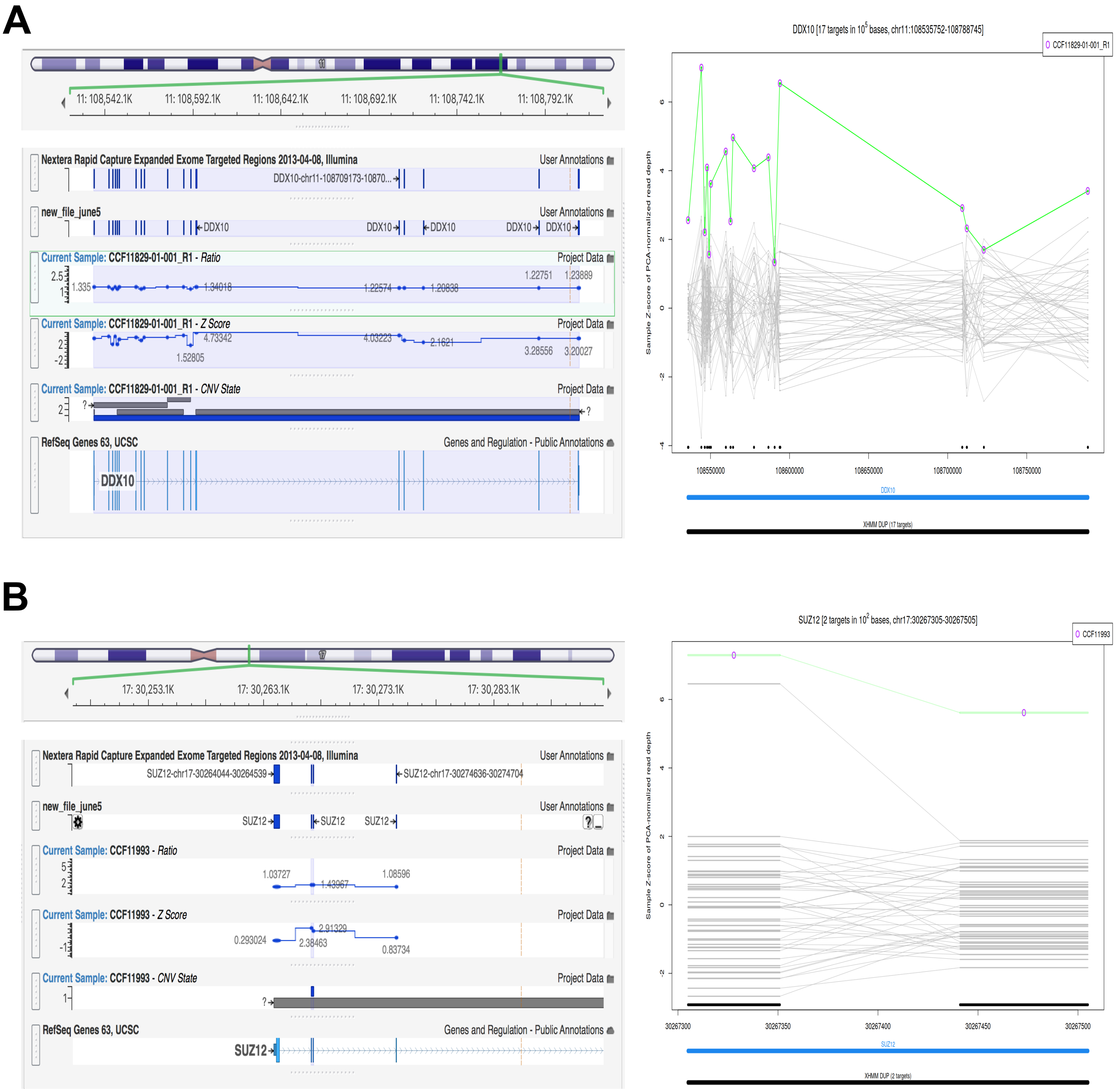

Supplementary Figure 3 Copy number variation analysis in CCF C-AYA cases. **a** *DDX10* duplication in a patient with osteosarcoma, VarSeq (left panel) and XHMM (right panel) platforms. **b** *SUZ12* duplication in a patient with Wilms Tumor, VarSeq (left panel) and XHMM (right panel) platforms. In VarSeq, Thick blue line in CNV state shows the duplicated area. In XHHM, green line is the target case, and all gray lines are other CCF cases in the series. Related to Figure 1.

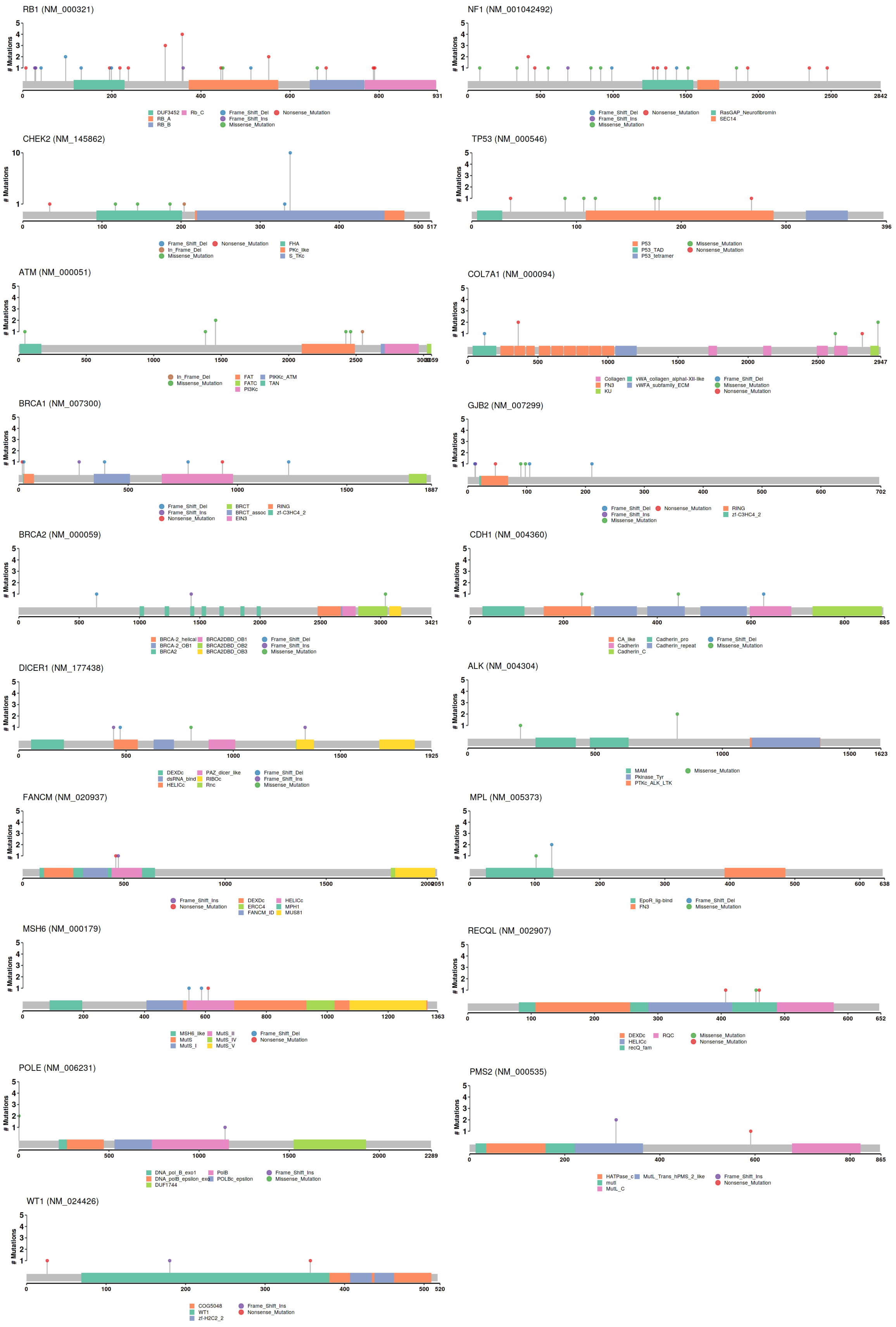

Supplementary Figure 4 Lollipop plots of top KCPG with germline P/LP variants in C-AYA patients with solid tumors. Related to Figure 2.

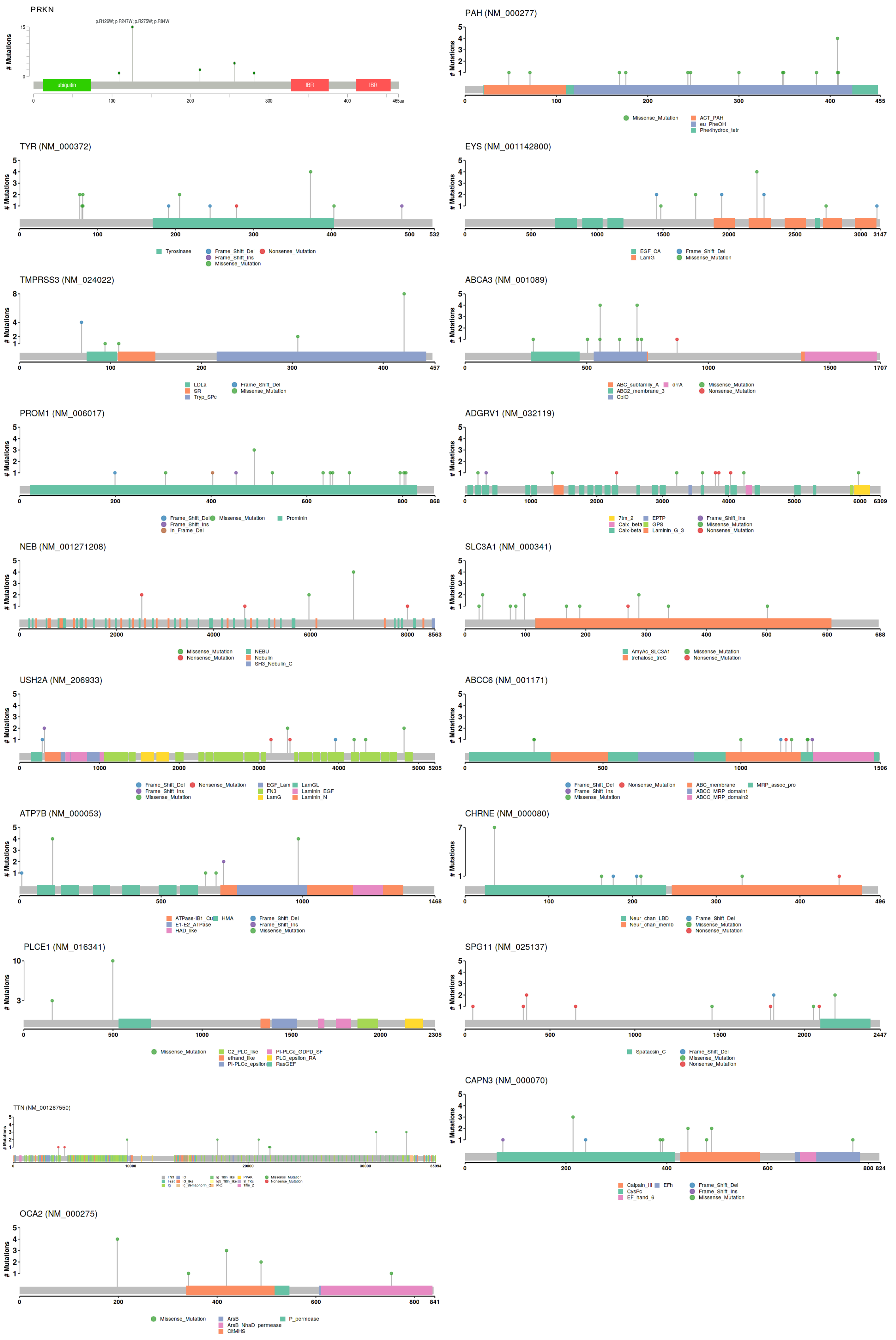

Supplementary Figure 5 Lollipop plots of top candidate genes with germline P/LP variants in C-AYA patients with solid tumors. Related to Figure 2.

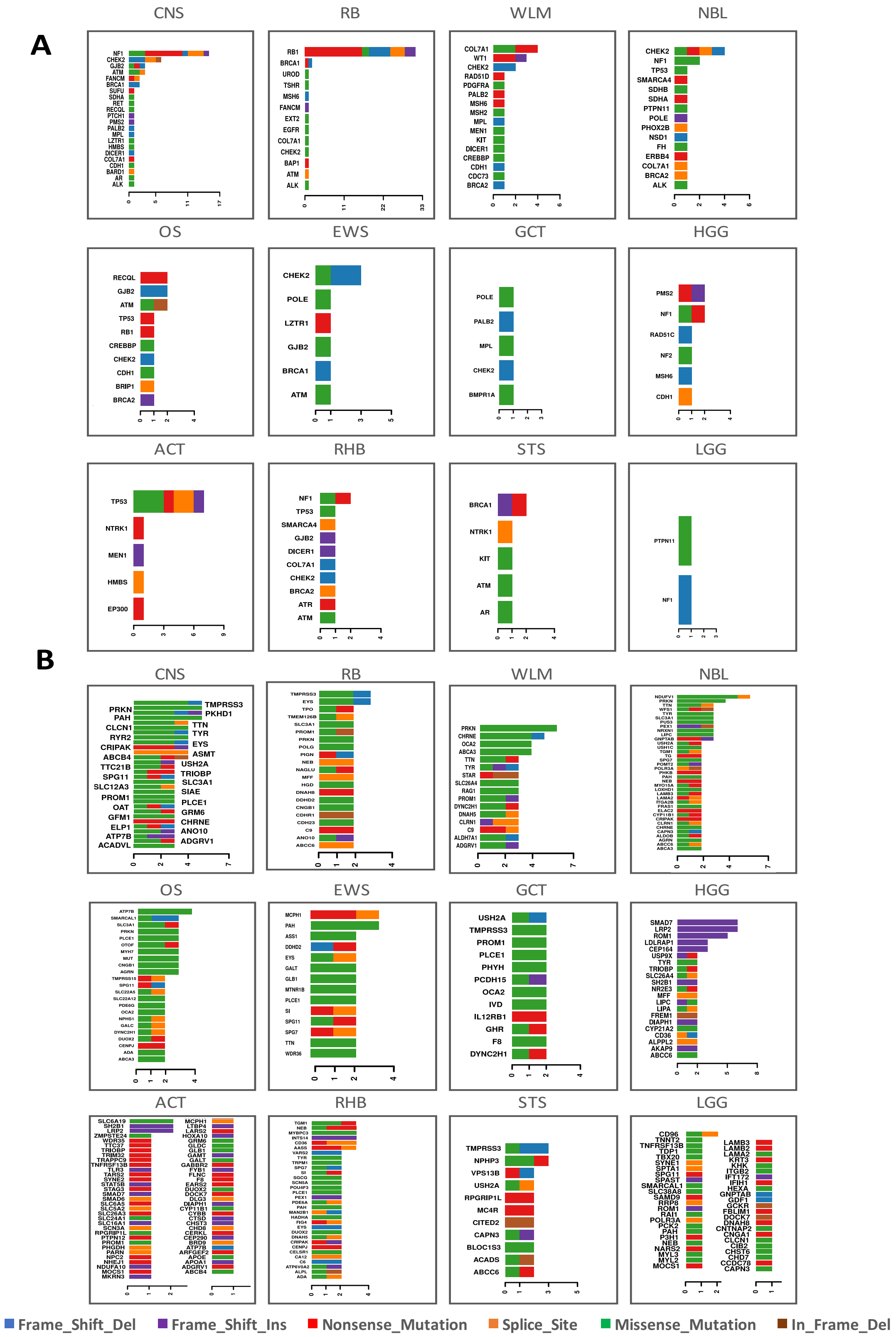

Supplementary Figure 6 Top genes with P/LP mutations in each C-AYA solid tumors. **a** Top altered KCPG genes in each tumor type. **b** Top altered candidate genes in each tumor type. Abbreviations: ACT, adrenocortical carcinoma; CNS, central nervous system; EWS, Ewing sarcoma; GCT, germ cell tumor; HGG, high-grade glioma; LGG, low- grade glioma; NBL, neuroblastoma; OS, osteosarcoma; RB, retinoblastoma; RHB, rhabdomyosarcoma; STS, soft tissue sarcoma; WLM, Wilms tumor. Related to Figure 3.

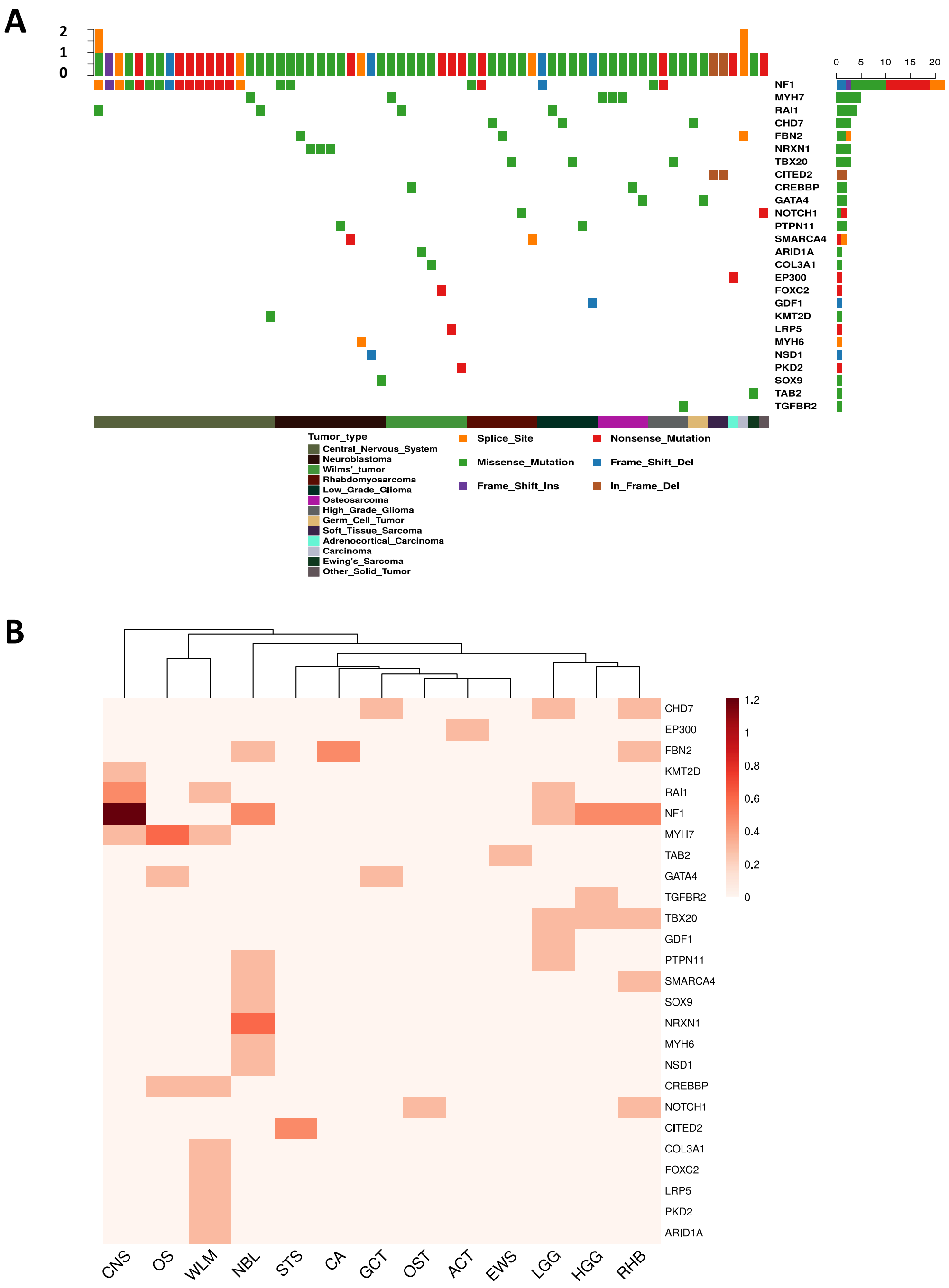

Supplementary Figure 7 Congenital heart defect (CHD) related genes with germline P/LP alterations in each C-AYA related solid tumor. **a** Oncoplots of CHD related genes . Each column represents one patient and its affected genes. **b** Heatmaps of CHD-related genes in each C-AYA related solid tumor. Scale refers to log10 (frequency of P/LP variants in specified genes in each tumor type). Abbreviations: ACT, adrenocortical carcinoma; CA, carcinoma; CNS, central nervous system; EWS, Ewing sarcoma; GCT, germ cell tumor; HGG, high-grade glioma; LGG, low- grade glioma; NBL, neuroblastoma; OS, osteosarcoma; OST, other solid tumors; RB, retinoblastoma; RHB, rhabdomyosarcoma; STS, soft tissue sarcoma; WLM, Wilms tumor. Related to Supplementary Table 6.

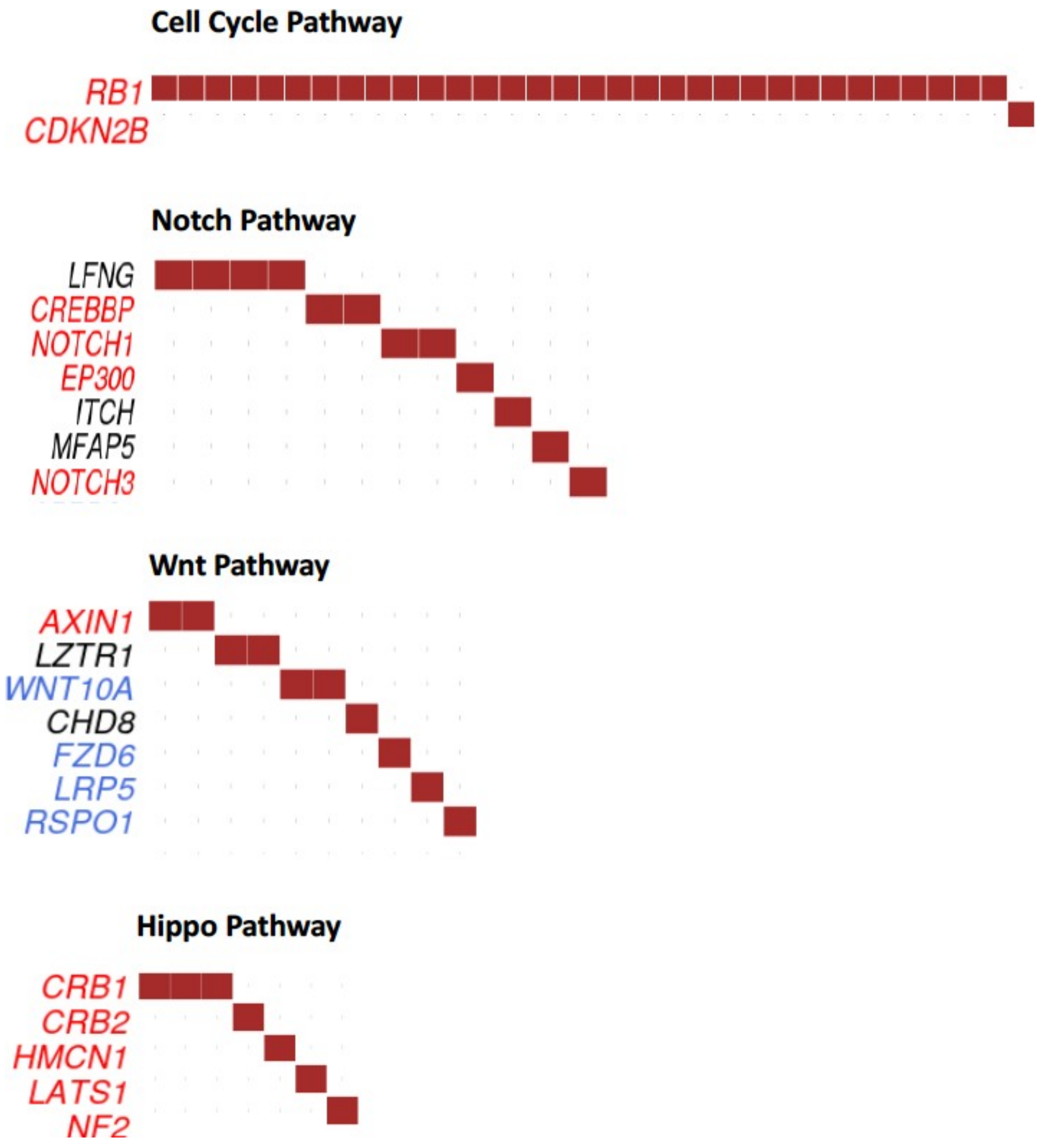

Supplementary Figure 8 Genes mutated in cell cycle, Notch, Wnt, and Hippo pathways, and the number of patients affected in our cohort. Red font: tumor suppressor genes; Blue font: oncogenes. Related to Figure 4.

Col7A1 Expression

KICH	Kidney Chromophobe
KIRC	Kidney renal clear cell carcinoma
KIRP	Kidney renal papillary cell carcinoma

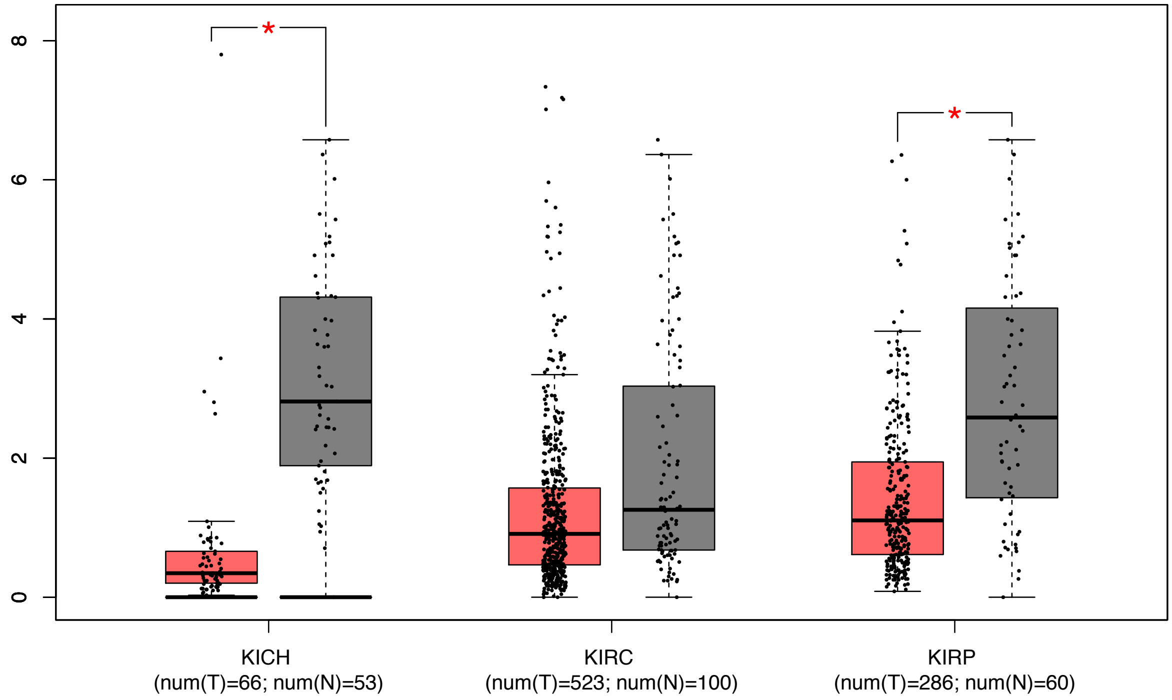

Supplementary Figure 9 Lower expression of *Col7A1* in kidney related tumors. Related to Figure 3.
Data extracted from gene expression profiling interactive analysis (GEPIA) website (Tang et al., 2017). log2(TPM + 1) have been used for log-scale. Line in the box shows median expression, top & bottom of the box are quartiles, and external lines show the range. Dots outside of the range are outliers. p-value Cutoff was 0.01 and Log2fc cutoff was 1.
