# Supplementary material for: Comprehensive germline genomic profiles of children, adolescents and young adults with solid tumors
Source: Nat Commun. 2020 May 5;11:2206. doi: 10.1038/s41467-020-16067-1 (PMC7200683; doi:10.1038/s41467-020-16067-1)
Supplement: Supplementary file 3 — Description of Additional Supplementary Files [file 41467_2020_16067_MOESM3_ESM.pdf]

Description of Additional Supplementary Files

Supplementary Data 1 Patients Demographics

Supplementary Data 2 List of known cancer predisposing genes (KCPG)

Supplementary Data 3 Pathogenic & likely-pathogenic variants in known cancer predisposing and candidate genes in CCF Series

Supplementary Data 4 Germline copy number variations in CCF C-AYA patients, confirmed by XHMM and VarSeq methods

Supplementary Data 5 Germline pathogenic & likely-pathogenic variants in known cancer predisposing genes of C-AYA patients with solid tumors

Supplementary Data 6 Evidence for pathogenicity & likely-pathogenicity of the variants in known cancer predisposing genes

Supplementary Data 7 Prioritized variants detected in autosomal-recessive known cancer predisposing genes

Supplementary Data 8 Patients with multiple pathogenic & likely-pathogenic variants in known cancer predisposing genes

Supplementary Data 9 Pathogenic & likely-pathogenic variants in candidate genes

Supplementary Data 10 Gene alteration summary of pathogenic/ likely pathogenic variants (known cancer predisposition genes + candidate genes)

Supplementary Data 11 Gene details of pathogenic/ likely pathogenic variants in known cancer-predisposition and candidate genes combined

Supplementary Data 12 Variant allele frequency comparison between C-AYA patients with solid tumors and non-TCGA ExAC dataset

Supplementary Data 13 Variant allele frequency comparison between C-AYA patients with solid tumors and non-TCGA ExAC dataset in each tumor type

Supplementary Data 14 Comprehensive table of known cancer predisposing genens detected in C-AYA solid tumors

Supplementary Data 15 Comprehensive table of known cancer predisposition genes detected in patients with C-AYA solid tumors

Supplementary Data 16 Congenital heart defect (CHD)-related genes found with germline P/LP variants in C-AYA patients with solid tumors

Supplementary Data 17 Pathways affected in C-AYA patients with solid tumors in association with germline P/LP variants

Supplementary Data 18 Top networks associated with pathogenic & likely pathogenic variants of C-AYA patients with solid tumors

Supplementary Data 19 Druggable genome and their related drugs info in both KCPG and candidate gene groups

Supplementary Data 20 C-AYA patients with solid tumor and their associated druggable genome
