# Supplementary material for: Comprehensive germline genomic profiles of children, adolescents and young adults with solid tumors
Source: Nat Commun. 2020 May 5;11:2206. doi: 10.1038/s41467-020-16067-1 (PMC7200683; doi:10.1038/s41467-020-16067-1)
Supplement: Supplementary file 24 — Reporting Summary [file 41467_2020_16067_MOESM24_ESM.pdf]

nature research

Corresponding author(s): Charis Eng, MD, PhD
NCOMMS-19-34930A

Last updated by author(s): 04/01/2020

Reporting Summary

Nature Research wishes to improve the reproducibility of the work that we publish. This form provides structure for consistency and transparency in reporting. For further information on Nature Research policies, see Authors & Referees and the Editorial Policy Checklist.

Statistics

For all statistical analyses, confirm that the following items are present in the figure legend, table legend, main text, or Methods section.

n/a	Confirmed	
☐	☒	The exact sample size (*n*) for each experimental group/condition, given as a discrete number and unit of measurement
☒	☐	A statement on whether measurements were taken from distinct samples or whether the same sample was measured repeatedly
☐	☒	The statistical test(s) used AND whether they are one- or two-sided<br>*Only common tests should be described solely by name; describe more complex techniques in the Methods section.*
☒	☐	A description of all covariates tested
☐	☒	A description of any assumptions or corrections, such as tests of normality and adjustment for multiple comparisons
☐	☒	A full description of the statistical parameters including central tendency (e.g. means) or other basic estimates (e.g. regression coefficient) AND variation (e.g. standard deviation) or associated estimates of uncertainty (e.g. confidence intervals)
☐	☒	For null hypothesis testing, the test statistic (e.g. *F*, *t*, *r*) with confidence intervals, effect sizes, degrees of freedom and *P* value noted<br>*Give P values as exact values whenever suitable.*
☒	☐	For Bayesian analysis, information on the choice of priors and Markov chain Monte Carlo settings
☒	☐	For hierarchical and complex designs, identification of the appropriate level for tests and full reporting of outcomes
☒	☐	Estimates of effect sizes (e.g. Cohen's *d*, Pearson's *r*), indicating how they were calculated

Our web collection on statistics for biologists contains articles on many of the points above.

Software and code

Policy information about availability of computer code

Data collection	Burrows-Wheeler Aligner (BWA v.0.6.1) (Li and Durbin, 2009)<br>http://bio-bwa.sourceforge.net/<br><br>Genome Analysis Toolkit (GATK 3.5) (Li et al., 2009)<br>https://software.broadinstitute.org/gatk/<br><br>Picard-2.18.1(Picard Toolkit, 2019) http://broadinstitute.github.io/picard/)<br>Ingenuity® Variant Analysis™ (IVA) software (version 5.4.20190121) Qiagen www.qiagenbioinformatics.com
Data analysis	MaxEntScan (Yeo and Burge, 2004)<br>http://hollywood.mit.edu/burgelab/maxent/Xmaxentscan_scoreseq.html<br><br>NHLBI ESP exomes (ESP6500SI-V2) Exome Variant Server http://evs.gs.washington.edu/EVS/<br>ExAC Frequency (0.3.1) (Lek et al., 2016)<br>http://exac.broadinstitute.org/<br><br>gnomAD Maximum Frequency (2.0.1) McArthur Lab (https://www.biorxiv.org/content/10.1101/531210v2)<br>https://macarthurlab.org/2018/10/17/gnomad-v2-1/<br>CADD (v1.3) (Rentzsch et al., 2019)<br>http://cadd.gs.washington.edu/info<br><br>SIFT prediction (2016-02-23) (Vaser et al., 2016)<br>https://sift.bii.a-star.edu.sg/<br><br>ClinVar (2018-08-01) (Landrum et al., 2016)

https://preview.ncbi.nlm.nih.gov/clinvar/variation/
Integrative Genomics Viewer (IGV) (Robinson et al., 2011; Thorvaldsdottir et al., 2013)
https://software.broadinstitute.org/software/igv/

Cancer Gene Census Germline 2019 (Sondka et al., 2018)
https://cancer.sanger.ac.uk/census#cl_search)
VarSeq™ v2.1.0 https://link.springer.com/protocol/10.1007%2F978-1-4939-8666-8_9 www.goldenhelix.com

eXome Hidden Markov Model (XHMM) algorithm (Fromer and Purcell, 2014)
https://atgu.mgh.harvard.edu/xhmm/

Maftools (Mayakonda et al., 2018)
https://bioconductor.org/packages/release/bioc/vignettes/maftools/inst/doc/maftools.html

Qiagen Ingenuity Pathway Analysis (IPA) (Kramer et al., 2014)
https://www.qiagenbioinformatics.com/products/ingenuity-pathway-analysis/

Allele Frequency Community (2018-09-06), RefSeq Gene Model (2018-07-10), PolyPhen-2 (v2.2.2), PhyloP (2009-11), DbSNP (151), TargetScan (6.2), GENCODE (Release 28), CentoMD (5.0), Ingenuity Knowledge Base (Stepford 190106.000), OMIM (May 26, 2017), BSIFT (2016-02-23), TCGA (2013-09-05), DGV (2016-05-15), COSMIC (v86), HGMD (2018.3)

For manuscripts utilizing custom algorithms or software that are central to the research but not yet described in published literature, software must be made available to editors/reviewers. We strongly encourage code deposition in a community repository (e.g. GitHub). See the Nature Research guidelines for submitting code & software for further information.

Data

Policy information about availability of data

All manuscripts must include a data availability statement. This statement should provide the following information, where applicable:

- Accession codes, unique identifiers, or web links for publicly available datasets
- A list of figures that have associated raw data
- A description of any restrictions on data availability

The Whole Exome data for C-AYA cases with solid tumors from Cleveland Clinic have been deposited in the NCBI Sequence Read Archive (SRA) database under the accession code PRJNA559601 (https://www.ncbi.nlm.nih.gov/Traces/study/?acc=PRJNA559601&o=acc_s%3Aa). Whole Exome data for C-AYA cases with solid tumors from St. Jude research hospital is accessible at https://www.stjude.cloud/ website. The non-TCGA data referenced during the study are available in a public repository from Broad Institute website at ftp://ftp.broadinstitute.org/pub/ExAC_release/release0.3.1/subsets/. All the other data supporting the findings of this study are available within the article and its supplementary information files and from the corresponding author upon reasonable request. A reporting summary for this article is available as a Supplementary Information file.

Field-specific reporting

Please select the one below that is the best fit for your research. If you are not sure, read the appropriate sections before making your selection.

☒ Life sciences ☐ Behavioural & social sciences ☐ Ecological, evolutionary & environmental sciences

For a reference copy of the document with all sections, see nature.com/documents/nr-reporting-summary-flat.pdf

Life sciences study design

All studies must disclose on these points even when the disclosure is negative.

Sample size	Solid tumors in C-AYA are very rare, so we started our study with 50 patients from Cleveland Clinic as a pilot study. To validate our findings with a larger independent series, we then analyzed germline exome data from 1,457 C-AYA patients with solid tumors from the St. Jude (StJ) dataset. 193 patients from the Pediatric Cancer Genome Project (PCGP) and 1269 patients from St. Jude Lifetime (SJLIFE). These were all the C-AYA solid tumor cases available for analysis from these two resources by that time. Considering the rare status of these tumors, 1507 cases were the highest number of C-AYA solid tumor analyzed for germline genomic signature by the time of the study.
Data exclusions	In order to eliminate those variants that commonly seen in general population and are not considered pathogenic or likely pathogenic,we implemented following established criteria : variants were excluded if the allele frequency was greater than or equal to 1.0% in any of the following population databases: 1000 genomes project (phase3v5b), NHLBI ESP exomes (ESP6500SI-V2), ExAC Frequency (0.3.1), and the gnomAD Maximum Frequency (2.0.1). Variants with a Phred-scaled CADD (v1.3) score <10 (http://cadd.gs.washington.edu/info) 46, or tolerant SIFT prediction (2016-02-23) were excluded as well unless there was an established pathogenic common variant.
Replication	As described above, we used an independent dataset from St. Jude hospital to reproduce the data from our pilot study on Cleveland Clinic series. All the analysis of this study performed multiple time to ensure the reproducibility of the findings.
Randomization	Our study did not include any intervention, so randomization was not applicable
Blinding	Our study did not include any intervention, so randomization was not applicable

Reporting for specific materials, systems and methods

We require information from authors about some types of materials, experimental systems and methods used in many studies. Here, indicate whether each material, system or method listed is relevant to your study. If you are not sure if a list item applies to your research, read the appropriate section before selecting a response.

Materials & experimental systems

n/a	Involved in the study
☒	☐ Antibodies
☒	☐ Eukaryotic cell lines
☒	☐ Palaeontology
☒	☐ Animals and other organisms
☐	☒ Human research participants
☒	☐ Clinical data

Methods

n/a	Involved in the study
☒	☐ ChIP-seq
☒	☐ Flow cytometry
☒	☐ MRI-based neuroimaging

Human research participants

Policy information about studies involving human research participants

Population characteristics

Our dataset included 1,507 patients with a median age of 6.41 ± 5.8 years consisting of 1,182 children (50.7% female, median of 5.2 ± 4.5 years), 164 adolescents (59.1% male, median age of 16.8 ± 1.3 years), 20 young adults (75% male, median age of 21 ± 2.4 years), and 141 unknown age-group who were diagnosed with solid tumors under 29 years of age. The most common tumor types included CNS tumors in 323 patients (21.4%), followed by Wilms tumors in 207 patients (13.7%), neuroblastomata in 190 patients (12.6%), and rhabdomyosarcomas in 134 patients (8.9%).

Recruitment

Patients' data for this project were obtained from two sources:
1. Cleveland Clinic Foundation (CCF): fifty patients initially diagnosed under 29 years of age with a solid tumor, presenting to the Pediatric Hematology-Oncology or the Cancer Genetics Clinics, were prospectively enrolled in this study under Cleveland Clinic-approved IRB protocol 8458. Final diagnosis and tumor types were confirmed by reviewing electronic medical records (EMR), including primary care physician notes, surgical notes, and pathological reports. Family history data and pedigrees were obtained by CCF genetic counselors. Any occurrence of related cancer in 1st and/or 2nd-degree relatives counted as a positive family history. Patients were evaluated for the occurrence of any relapse, metastasis, second primary malignant neoplasm (SMN), or death, which collectively we classified them as patients with high-burden tumors.

2. St. Jude (StJ) cloud: The rest of the patients' germline/clinical data were obtained from two datasets within the St. Jude Cloud, generated by St. Jude Children Research Hospital and McDonnell Genome Institute of Washington University School of Medicine, under legal agreement 4147653:
a. 193 patients from the Pediatric Cancer Genome Project (PCGP)
b. 1269 patients from St. Jude Lifetime (SJLIFE)

Ethics oversight

Cleveland Clinic-approved IRB protocol 8458
t. Jude cloud under legal agreement 4147653

Note that full information on the approval of the study protocol must also be provided in the manuscript.
